# Relapses of bipolar/cycloid psychosis related to childbearing

**DOI:** 10.1007/s00737-016-0682-9

**Published:** 2016-10-13

**Authors:** Ian Brockington

**Affiliations:** University of Birmingham, Edgbaston, Birmingham, B15 2TT UK

**Keywords:** Multiple relapses, Early postpartum trigger, Prepartum psychosis, Multiple relapses

## Abstract

It was observed nearly 200 years ago that mothers with puerperal psychosis may recover, then relapse, sometimes repeatedly. This phenomenon seems to be better recognized in the American and French literature, where it has been reported in a large minority, or even majority, of cases. It offers an opportunity to study the pathogenesis of psychosis.

## Introduction

The tendency to recurrence is the earliest, best-established and most widely known fact about of non-organic postpartum psychosis. But relapses (recurrences not more than 2 months after recovery from an episode) are also common and one of its most interesting features—an ancient observation that has been almost completely ignored.

It was first described almost 200 years ago (Esquirol 1816):

A 23-year old suffered from nervous disorders from her menarche. At 21 she married a general, and promptly became pregnant. Seven days after the birth, there was an adverse event. Her lochia were suppressed and she started to rave, but for only 2 days. She relapsed on the 25th day. *Délire furieuse* switched to stupor. In the 5th month her periods returned and she became obese. In spite of the death of her husband, she remained well.

This case is atypical in the extreme brevity of the 1st episode. Another case he described (Esquirol 1819), with four triggers, also suffered a relapse after recovery from her weaning episode:

A 26-year old gave birth to her 1st child. On day 3 she developed furious mania for 2 months. Every spring she showed exaltation without *délire*. At 30, weaning her second child aged 1 year, she developed furious mania, from which she recovered soon after hospitalisation; but, 2 days after her discharge, she relapsed, recovering after 3 months. At 34 she had a 2-month miscarriage; the next day she became loquacious and developed furious mania that lasted only a few days.

## Relapses in the literature

### Exclusions

In the enumeration of cases, I excluded the following:

- The recurrence of a prepartum episode after the birth
- Unusual cyclical disorders (Brockington 2014)
- Runge psychoses (Runge 1911)
- Organic psychoses
- Switches from mania to depression or vice versa in a continuous illness
- Deterioration explained by an adverse event such as seizures or psychological trauma.

### Enumeration

After these subtractions, at least one relapse was reported in 289 cases, with the following onsets, related to the episode totals of each onset group:

- Post-abortion 3 (3 %)
- Prepartum 9 (3 %)
- Early postpartum 145 (11 %)
- 4–13-week onset 37 (9 %)
- Late onset 13 (6 %)
- Weaning onset 3 (8 %)
- Unknown onset 19 (5 %)

The nine prepartum cases included five with the relapse before the birth (Dubrisay 1858, Bierende 1920, Mares and Barre 1962, Wise et al. 1984, Luauté et al. 1991). Three followed the postpartum continuation of a prepartum psychosis, so were in fact postpartum. There was the following Argentinian case (Lértora 1955):

A woman, whose father committed suicide, had a 3-month miscarriage in 1938, and normal births in 1940 and 1944. In 1950 she became clouded in the last month of pregnancy, with poor memory for the birth. In February 1953 she again became clouded, excited and disorientated during pregnancy, this time in the 5th month. She responded to ECT, but relapsed. She gave birth in June and remained confused during the first month of the puerperium, recovered with ECT, then relapsed, with clouding of consciousness, disorientation, insomnia and incoherence. She responded to cortisone treatment.

This patient had a prepartum psychosis (2nd trimester onset) with a relapse, continuing into puerperium, also with a relapse.

In addition to Esquirol’s case, two mothers with weaning onsets had relapses (Marcé 1856, Bourson 1958); Marcé’s patient had several relapses.

### Multiple relapses

There were 23 episodes with two relapses and 15 with three or four, while 27 episodes were followed by multiple relapses of uncertain number or the description of a periodic, phasic, alternating or remittent course. Ten mothers with six or more relapses all had evidence of a menstrual association. Several mothers had relapses following episodes associated with two separate births (Warburg 1915, Fauré-Amiel 1962, Moisan 1982).

### Knowledge of this phenomenon

It is curious that this phenomenon is hardly ever mentioned. To explore the reason for this, case reports were studied by epoch and language group. The number in each generation is shown in Table 1.

**Table 1:** Reports of relapses by generation

Years	Episodes with relapses	Non-organic postpartum psychoses	Percentage
1801–1825	2	43	5
1826–1850	3	73	4
1851–1875	14	135	10
1876–1900	10	305	3
1901–1925	23	391	6
1926–1950	38	522	7
1951–1975	73	408	18
1976–present	25	245	10
Total	188	2122	8

The increase since 1951 is best explained by increased awareness of the phenomenon, but there are differences between different language groups, as shown in Table 2.

**Table 2:** Reports of relapses by language group

Language	Cases with relapses	Non-organic postpartum psychoses	Percentage
French	85	715	12
American	26	224	12
Italian	18	198	9
Other	20	214	9
German	26	472	6
British commonwealth	8	211	4
Dutch	3	88	3

The lowest rates were reported by authors from the Netherlands and the British Commonwealth. Two large Dutch series (Van Steenbergen-van-der-Noordaa 1941, Visscher 1949), with a total of 61 non-organic postpartum psychoses, contributed only two cases with relapses. In contrast, high rates were reported by American authors, one of whom (Bower and Altschule 1956) reported that 17/39 (44 %) had at least one relapse. Almost half the reported cases are in the French literature, of which 55 were reported by six authors (Balduzzi 1951, Sivadon 1933, Delay et al. 1948, Bourson 1958, Cain et al. 1959, Fauré-Amiel 1962) with rates as high as 50 % (Delay et al. 1948) and 73 % (Cain et al. 1959). Cain’s eight cases are briefly summarized in Table 3 and Delay’s ten cases in Table 4.

**Table 3:** Relapse of postpartum psychosis: series published by Cain et al. 1959

Clinical details	Onset of psychosis	Recovery	Relapse
Case 2: early onset of “hallucinatory delirium” with logorrhoea and ideas of her own death and that of her mother	Day 10	After 5 ECT	Day 25 requiring five more ECT
Case 4: early onset of agitation with confusion and disorientation. Alternating manic excitation and anxious indifference	Day 3	With ECT	The same symptoms requiring more ECT
Case 7: early onset of excitement with incoherence, agitation, delusions of guilt and erotomania. Alternating excitement and stuporose confusion	Day 3	With ECT	Relapse, no details
Case 8: confusion and incoherence, then melancholic stupor with euphoria, flight of ideas and logorrhoea	Day 3	With ECT	Relapse after a week, recovery, 2nd relapse 2 weeks later
Case 12: agitation, logorrhoea, incoherence, ideas of persecution by her husband, and morbid jealousy	One month after the birth	With ECT	Three relapses
Case 13: psychosis with polymorphic delusions and extreme excitement	Some days after the birth	Rapid response to ECT	Relapse, no details
Case 15: manic excitement with mental confusion	One week after the birth	After nine ECT	Relapse a week later, requiring more ECT
Case 16: stupor, with mutism, food refusal, agitation with auto-accusation, cursing and hating everyone, violence	Day 19	With ECT	One relapse, no details

**Table 4:** Relapse of puerperal psychosis; series published by Delay et al. 1948

Details of case	Course
Case 1: on day 9, onset of a variable polymorphic state with alternation of depression and poorly systematized delusions of persecution.	Recovery after 20 ECT, relapse with mutism, catatonia, delusions and excitement, requiring more ECT, followed by three more relapses at intervals of 2 months and 3 weeks, requiring further treatment by insulin comas and ECT.
Case 4: on day 8, she was hospitalized with depression and ideas of cancer; she was disorientated and could hear people speaking to her and the cries of her mother suffering torture. Excitement alternated with stupor and catalepsy.	After nine ECT, her mind cleared. On day 43, her menses appeared, and on day 48, she relapsed and required 15 more ECT.
Case 5: onset 1 month after the birth: she misidentified people and had hallucinations of people talking all the time. Hospitalized over 4 months later, she was disorientated and had visual and auditory hallucinations. She became excited and, 6 months after the birth, received ECT.	She improved within 2 weeks but relapsed a few days later. ECT was resumed. A state of excitement continued with persecutory ideas, and she was treated with insulin comas.
Case 7: on day 9, she became excited—laughing, crying and talking incoherently. On day 19, she was hospitalized—disinhibited, euphoric, shouting and dancing.	With 8 ECT, she improved. About a week later, she relapsed, recovered and was discharged 5 months after the birth.
Case 9: 2 months after the birth, she became disturbed and, after 4 months, was admitted to hospital with excitement, fugues, singing, constant joking, flight of ideas and complete insomnia.	Two weeks later, after the reappearance of her menses, she calmed down. A month later, she relapsed. After ECT, she was discharged 8 months after the birth.
Case 11: on day 6, she began to say that her husband wanted to get rid of her; she was being poisoned and persecuted by an unknown group. She was sleepless and refused food.	Within a month, after five ECT, she improved. In the 6th week, her menses appeared, and she relapsed with delusional depression, requiring another five ECT.
Case 12: on day 10, she became manic with flight of ideas, confusion, ideas of persecution and auditory hallucinations.	At 3 weeks, she improved. In the 2nd month, she relapsed, with confusion and food refusal. With 12 ECT, she improved, but, at 3 months, relapsed again. With more ECT, she improved but relapsed 8 months after the birth.
Case 15: on day 7, she became manic, with shouting, singing, familiarity and exuberance.	Treated with ECT, she improved. Her menses returned at 2 months, preceded by a relapse. She had several phases of excitation. She recovered 9 months after the birth.
Case 16: after a birth complicated by a fever of 40°, she became agitated, then sank into stupor, mutism and prostration. She claimed that her own child was dead, and this one was not hers.	Treated with ECT, she lost her delusions. Five months after the birth her menses appeared, with a relapse of melancholic delusions and food refusal. She recovered with further ECT.
Case 20: within a week of the birth, she became depressed with delusions of guilt.	Treated with ECT, she improved, but a month later relapsed with excitation. After 25 insulin comas, she recovered.

Differences between language groups can only be explained by under-reporting or over-reporting, and the first is more likely. Dutch, British and, to some extent, German authors were either ignorant of this phenomenon or undervalued its importance. If the French observations are accurate, the frequency is much higher than is evident from the general literature.

### Relapses in my series

Relapses were noted in 74 mothers, of which 61 were in the bipolar/cycloid group and 13 among the others (*p* = .012) (Brockington 2014). The high figure in the bipolar/cycloid group (28 %) is underpinned by the much higher rates found in several French studies. Focusing on this group, the great majority followed an early postpartum episode with a frequency of 62/296 episodes (21 %): there was one after a post-abortion episode, two after 4–13-week onsets and two with unknown onset. Multiple relapses were noted in 24 mothers—12 relapsed twice, eight thrice and one each four, five and six times. Five had relapses after two separate postpartum episodes, including two with different onsets—one with a post-abortion and early postpartum onset and one with a 4–13-week and early postpartum onset. Two had relapses after each of three early postpartum episodes.

## Discussion

During the last 50 years, clinicians might be tempted to attribute a relapse to premature reduction of medication or poor compliance. But only 116/223 cases have been published since 1953, when chlorpromazine was introduced as a treatment for psychosis. Since non-compliance *cannot* be the explanation for many or most of these cases, one must find another. An explanation that springs to mind is the influence of menstruation. Until someone comes up with another reason for periodic relapses weeks and months after childbirth, this is the only hypothesis in the field.

It is obvious from Esquirol’s first case and many others that episodes followed by a relapse are distinguished by the brevity of the first phase. Although their total length was longer (median 96 versus 46 days), the first phase had a median of only 25 days, with 19 lasting less than 3 weeks (Brockington 2014). This raises the possibility that relapsing cases are a distinct variant of early onset postpartum psychoses. This is disproved by the fact that, in my series, 18 mothers had episodes of both kinds. The explanation must, therefore, be that the brevity of the first phase, with an interval of health, *allowed the relapse to be observed*. Unless there was a complete recovery, the clinician would not notice the phasic course. Thus, although a phasic course is common, and not due to treatment failures, it is probably even more common than the number of relapses suggests.

The relapsing tendency offers an opportunity for research into the pathogenesis of psychosis, similar to that conducted in recurrent puerperal and menstrual psychosis (Brockington 2016). Two groups (Wieck et al. 1991, Meakin et al. 1995) have studied pregnant women with a history of puerperal psychosis, focusing on the puerperium after a subsequent birth. This strategy is feasible but involves a search for pregnant women at high risk. Menstrual psychosis has the advantage of more frequent recurrences, but its frequency in the general community is only about 1/10,000 and a minority has a persistent long-term periodic disorder. Those working on mother-and-baby units, however, admit 10–20 women each year with acute puerperal psychosis. About a quarter of these will have at least one relapse, and one or two each year will have multiple relapses. Without funding, consultants can accumulate evidence on the causal factors and pathogenesis of these relapses.
